# Arogyasree: An Enhanced Grid-Based Approach to Mobile Telemedicine

**DOI:** 10.1155/2010/536237

**Published:** 2010-05-11

**Authors:** Sriram Kailasam, Santosh Kumar, Janakiram Dharanipragada

**Affiliations:** Department of Computer Science & Engineering, Indian Institute of Technology Madras, Chennai 600036, India

## Abstract

A typical telemedicine system involves a small set of hospitals providing remote healthcare services to a small section of the society using dedicated nodal centers. However, in developing nations like India where majority live in rural areas that lack specialist care, we envision the need for much larger Internet-based telemedicine systems that would enable a large pool of doctors and hospitals to collectively provide healthcare services to entire populations. We propose a scalable, Internet-based P2P architecture for telemedicine integrating multiple hospitals, mobile medical specialists, and rural mobile units. This system, based on the store and forward model, features a distributed context-aware scheduler for providing timely and location-aware telemedicine services. Other features like zone-based overlay structure and persistent object space abstraction make the system efficient and easy to use. Lastly, the system uses the existing internet infrastructure and supports mobility at doctor and patient ends.

## 1. Introduction

The European Commission's health care telematics programme [1] defines telemedicine as “rapid access to shared and remote medical expertise by means of telecommunications and information technologies, no matter where the patient or relevant information is located.” Telemedicine has prime importance especially in developing countries because of several reasons. The primary health care providers in the rural areas are mostly inexperienced [2]. In India, about 70% of the population live in the rural areas while 75% of the doctors practice in the urban centers [3, 4]. Overall, there is only one doctor for every 15 500 people. However, the telecommunications infrastructure in India is continuously improving [2]. All the above-mentioned facts make telemedicine an appropriate technology for developing countries, particularly India. 

The last decade has seen the emergence of many telemedicine setups all around the world [5, 6]. A typical system involves a small set of hospitals providing remote healthcare services to a small section of the population using satellite technology, nodal centers, mobile health units, and so forth. However, we visualize the need for much larger Internet-based telemedicine systems. Large-scale systems enable a large pool of doctors and hospitals to collectively provide healthcare services to entire populations. Patient records can be made accessible to doctors from any location seamlessly. This significantly increases a patient's chances of receiving high quality care even when specialist doctors are not available nearby. A large-scale telemedicine system is more capable of supporting increasing numbers of patients and is more robust against failures and attacks. 

Now, central server-based systems have single point of failure and cannot scale to huge number of requests. This was confirmed by a survey conducted by Intel (one of the project sponsors) of the telemedicine infrastructure at Narayana Hrudulaya (NH), a world-class cardiac hospital. The NH telemedicine infrastructure is supported by the Asia Heart Foundation and the Indian Space Research Organization (ISRO) to provide satellite connectivity between Bangalore and several parts of India and even neighboring countries. A nodal centre is created in a local hospital in these remote areas where doctors and patients can directly interact with specialists at NH. The remote ECG unit can either directly dial into the NH server or access the Internet and send its request to the NH server through an FTP interface. On an average day, 80 ECG reports are received and processed at NH. However, as the number of telemedicine units' increase, the NH centre at Bangalore becomes a bottleneck. Besides, the doctor must be physically present at the hospital centre to deliver responses promptly. This requirement (of doctor's physical presence at the hospital centre) hampers the number of doctors that can provide service. Thus we conclude that a scalable solution must not be based on a central server and it must facilitate specialists to provide service from wherever they are. 

Recent research [7] has shown P2P systems to be scalable and robust. Hence, we envision a large-scale telemedicine solution consisting of multiple hospitals (across the country) acting as peers. As of now, there is no dedicated infrastructure connecting all the hospitals in the country. Hence, we propose to support telemedicine services with P2P technologies using the internet as the backbone. This will facilitate cost-effective utilization of the existing hardware resources and in future (if at all) an easy integration with dedicated infrastructure. The recent advances in broadband technology for mobile phones add a new dimension to telemedicine by facilitating any time-anywhere computing. Now, the specialist located anywhere could use his mobile device to access the patient reports via the Internet and provide advice. With WiMax/GPRS capable of providing cheap Internet access in rural areas, it is possible to provide mobility support even at the patient's end. This will enable a nurse-led mobile telemedicine unit and lead to better coverage in the rural areas. 

**Figure 1:**
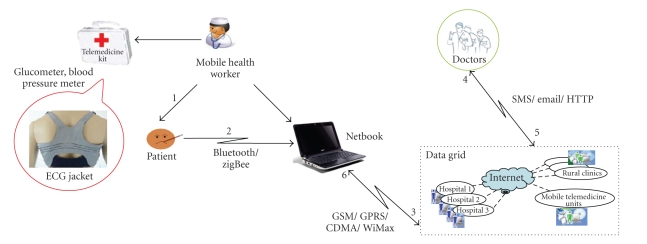
Life cycle of a patient request in Arogyasree.

In this paper, we present Arogyasree (*Arogyasree* means “good health” in Sanskrit), a context-aware, P2P grid framework for mobile telemedicine. This Internet-based scalable system integrates multiple hospitals, mobile medical specialists and rural mobile units/clinics to form a large virtual enterprise. The current version of the telemedicine system is based on the store and forward model—the bio-medical signals of the patient are stored in the data grid and then scheduled to an appropriate doctor. Figure 1 shows the high-level view of the proposed telemedicine system. 

Health workers carry portable instruments for measuring vital health parameters such as blood pressure, ECG jacket, blood sugar, and weight. These devices are wirelessly connected with a Smartphone/PDA, which uploads the data into the grid. The context-aware grid scheduler matches the request context to the nearest available doctor and notifies him via email/SMS. The doctor then makes an evaluation of the measured data and provides consultations by telephone/Internet. 

This paper makes the following contributions. First, it discusses the design of a distributed context-aware scheduler (using tuple spaces) that considers parameters like proximity, patient history, severity of ailment, and so forth to locate appropriate doctors. Many existing telemedicine solutions have overlooked the problem of context-aware scheduling of patient requests or have addressed it minimally. Attempts like [8] to integrate vital sign sensors into textiles (to continuously monitor the patient) will generate huge amount of data, and hence locating an appropriate doctor becomes important. Second, the system provides an easy-to-program abstraction for building applications. Basically, it supports a persistent object-space abstraction for the storage of medical data on the grid. Third, it uses a unique-zone based overlay structure (Vishwa [9]) that ensures proximity in routing as well as storing data. The proximity-aware overlay structure and the scheduling mechanism minimize the data movement cost and improve the efficiency of the system. Finally, the solution uses the existing internet infrastructure and supports mobility at doctor and patient ends. All the above features sum up to make Arogyasree an appropriate architecture for large-scale P2P grid framework for telemedicine.

The remaining of this paper is organized as follows. Section 2 discusses the related work while Section 3 explains the system model. Section 4 discusses the application scenarios, the implementation details, and limitations of the proposed system. Section 5 presents the performance results, and we conclude in Section 6.

## 2. Related Work

We compare Arogyasree with the existing telemedicine solutions with respect to scalability, context-aware scheduling of patient requests, coverage of the system (mobility enhances coverage of the system), telemedicine services, infrastructure (dedicated versus nondedicated), and so forth. 

Telemedicine solutions such as [5, 6, 10, 11] use dedicated hospital nodes as centers for providing telemedicine service. They require the specialist to be available at the centre whenever the data is received to ensure that the advice is sent within few minutes. But with the widespread use of mobile communication devices, the reports can be delivered on to the handheld of a specialist, who may be located, anywhere. Arogyasree supports mobility in the part of the medical practitioner like in [12]. It also supports mobility in the patient side thereby improving the coverage in rural areas. Solutions like [13–15] also support mobility in the patient side. In [14], they use wireless ad hoc networks and grids at the patient side. However we have modeled the patient-side resource-constrained mobile device as external entities (not as peers in the grid) to improve the grid stability. Certain solutions address the need for a single hospital and associated patients as in [12]. But our solution incorporates multiple hospitals working collaboratively within the grid providing a farther reach to the target community. Solutions like [16, 17] also use interconnected hospitals to provide medical service. Both these solutions, however, do not support mobility on the doctor side as they use video conferencing as a primary mode of patient-doctor interaction. Additionally, [17] requires dedicated communication infrastructure linking the hospitals whereas our solution is built on top of the Internet.

In [11, 15], a central server is used to handle the incoming requests. This makes them inherently nonscalable restricting their scope as a solution for a large-scale global health grid. Also, the central server becomes a single point of failure. Our solution uses multiple zonal servers from various zones to distribute the handling of requests, thereby providing scalability. The solution in [10] uses a different approach for the above problem by using a dedicated server farm to provide request handling. But this leads to underutilization of the computing resources of the nodes present in various hospital nodes and limits scalability. The solutions also differ in the manner in which patient requests are forwarded to the doctors. In [14], a dedicated medical call center comprising of medical practitioners attend to patient requests and forward them to appropriate doctors. But the manual intervention in forwarding the requests makes the system expensive as well as less scalable. In another approach [18], the system accepts the symptoms from the patient and uses artificial intelligence to route it to the appropriate specialist. In our solution, we used a simple automatic forwarding of the requests through the zonal servers based on the specialization as specified in the request. Our solution also allows internal forwarding of the patient requests among multiple specialists. Thus we have achieved a simple, scalable, and cost-effective solution. Solution in [18] does not address the need of a patient-doctor meeting as its primary aim does not require it (military application). But in our case the patient may need to consult the doctor in person depending on the ailment. Hence scheduling is implemented in a way that (as far as possible) the patient requests will be forwarded to the nearer hospitals depending on availability. For this, the presence of geographic proximity-based zones and local tuple spaces for handling surplus requests is used in our solution. 

Many of the solutions utilize medical grids for computational purposes only as in [19, 20]. But we have also used the grid for storage of patient records, request forwarding, and load balancing. Further, none of the above solutions effectively address the possibility of a group of hospitals becoming overloaded occasionally. This requires the request to be stored temporarily to ensure proximity and later forwarded to the global grid after a specified time-out to ensure timely service. This has been addressed by incorporating locationwise and global tuple spaces. 

## 3. System Model

In this section, we present the architecture of Arogyasree, a large-scale P2P data grid framework for telemedicine. We primarily focus on the overlay structure, persistent object-space abstraction, context-aware scheduling, and storage management. The other details are available in [21].

### 3.1. System Overview

The telemedicine system makes the following assumptions.

- The nodes are contributed by the participating hospitals. Every node shares a minimum amount of storage space as per an agreement signed by all participating hospitals.
- The administrative policies governing these nodes are aligned as per a common agreement between the hospitals. This is feasible since there is no large-scale computerized patient health record management in India [22] and this is a unique opportunity to build one.
- The doctor's and the health worker's mobile devices have Internet connectivity.

The telemedicine system classifies resources into two types: data storage resources (nodes contributed by hospitals) and medical resources (doctors with mobile device). The data storage resources are modeled as peers in the telemedicine grid while the medical resources are modeled as external entities which contact or are contacted by the grid nodes for providing/accepting services. The data grid provides a persistent object-space abstraction that allows us to model real-world entities like patients, doctors, and so forth. as objects and is discussed in Section 3.3.2. We use a unique zone-based overlay structure (a zone is formed by grouping geographically closer nodes) like in Vishwa. The overlay ensures proximity in routing as well as data storage. The details of the overlay are discussed in Section 3.2. The data of patients belonging to the geographical region covered by the zone is replicated on nodes within the zone to ensure replica proximity (efficient updates to data). Few replicas are also maintained in other zones to ensure data availability in case the entire zone gets disconnected. Since the patient data can be distributed on the grid, the system provides clustering mechanisms to enable efficient retrieval of patient history and is discussed in Section 3.3.3.

The nodes in the telemedicine system assume different application roles based on requirement. The application roles are zonal server, hospital representative, and grid node. The zonal server acts as patient request gateway or entry point for the patient requests. The hospital representative (per hospital) provides an interface to the doctors to indicate their availability. It also periodically advertises the aggregate availability information (of doctors) along with a list of capable nodes to the zonal server (henceforth, referred as resource advertisement). The idea is that the capable nodes in terms of CPU and memory utilization (at that point of time) will be used to serve the patient requests. The grid nodes are nodes other than the zonal server and are used for storage and computation. 

We propose a distributed context-aware resource discovery mechanism to schedule the requests as centralized schemes do not scale with the increasing number of requests/resources and have a single point of failure. In our scheme, the zonal server (request gateway) accepts the location coordinates and the area of ailment and redirects the request to one of the capable nodes advertised by a proximal hospital within the zone. That node receives the patient request object (the upload bandwidth of the capable node is used to accept patient request file and not the zonal server's). It considers context parameters like patient history, language, severity of ailment (emergency/normal), and so forth and selects an appropriate doctor from its local resource information for scheduling. If there is none available, then tuple spaces are used for temporary storage and forwarding as discussed in Section 3.4.2. 

**Figure 2:**
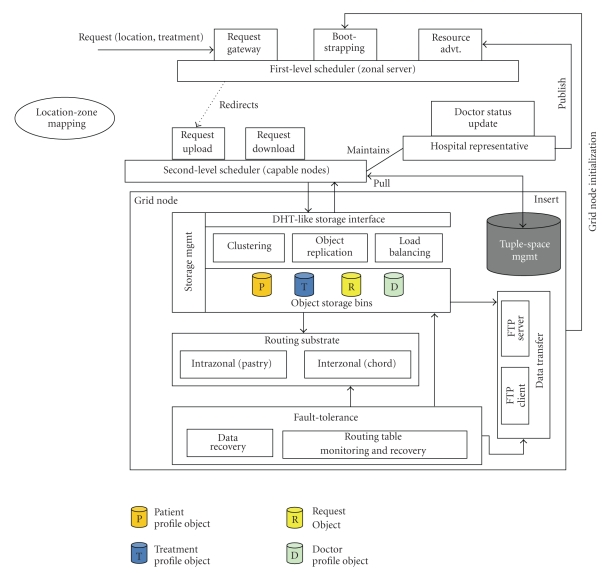
Architecture of telemedicine grid.

Figure 2 shows the architecture diagram. The details are discussed in the following sections.

### 3.2. Overlay

**Figure 3:**
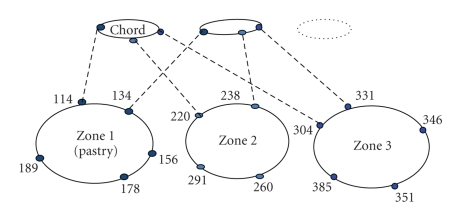
Overlay structure.

The telemedicine grid uses a zone-based overlay. Roughly, a zone spans across a state or a group of cities depending on the population of the region, the number of hospitals in that region, and so forth. Thus proximal nodes are part of the same zone. As seen in Figure 3, each node participates in two overlays, viz., Pastry [23] within the zone and Chord [7] across zones. Both Pastry and Chord are DHT-based routing substrates and provide lookup guarantee of *O*(log *N*) hops, where *N* is the number of nodes in the overlay. 

We make the following three observations from Figure 3.

- The number of nodes in a single Pastry overlay is equal to the number of nodes in the zone.
- The number of nodes in the Chord overlay is equal to the number of zones.
- Each node is part of an independent Chord ring across zones.

Thus, if we assume that the total number of nodes in the entire system is *N*, the number of zones is *Z*, and the average size of each zone is *K*, then the routing table size per node in our overlay is

(1) $$\begin{array}{c} \text{Pastry}\;\text{routing}\;\text{table}\;\text{size}=O\left(\log\;\;K\right),\\ \text{Chord}\;\text{routing}\;\text{table}\;\text{size}=O\left(Z\right). \end{array}$$

Hence, total routing table size per node ~(log  *K* + log  *Z*) = log (*K*∗*Z*) = log (*N*), which is the same as that of a single large overlay. The benefits of such an overlay structure are as follows

- Intrazonal routing (i.e., message routing between nodes belonging to hospitals within the same zone for replication or scheduling) does not pass through nodes outside the zone, thereby saving bandwidth and ensuring proximity in routing
- Interzonal routing: the message is first routed to the closest node in the same zone using Pastry and then to the destination node using the Chord finger table of the closest node. Thus efficient routing is facilitated. More details are available in [9].

In conclusion, this overlay structure ensures efficient routing. Besides, prefix-based routing in Pastry is exploited for clustering the request objects of a patient (patient history) and load balancing as explained in Section 3.3.3. Chord is used across zones because it ensures correctness in routing even if very few routing table entries are correct (lazy maintenance for the Chord neighbors across zones).

### 3.3. Storage Management

Storage management aims at clustering the patient data for fast retrieval while simultaneously maximizing the storage utilization of the grid (nodes may have heterogeneous storage capacities). It also ensures high availability by replicating the objects within as well as across zones. 

#### 3.3.1. Object Replication

Every object to be stored on the grid is assigned a unique identifier (by prefixing the zone id to a quasirandom identifier generated using SHA-1). The object replicas are stored on *k*-closest nodes (in the id space) within the zone (Pastry). Proximity while storing data replicas enables efficient propagation of updates among them. The object is also replicated on “c” successors in the Chord ring to handle disconnection of the entire zone in the event of network outages/disasters. The system also supports on-demand replication (for optimization based on the access pattern).

#### 3.3.2. Types of Storage Objects

**Figure 4:**
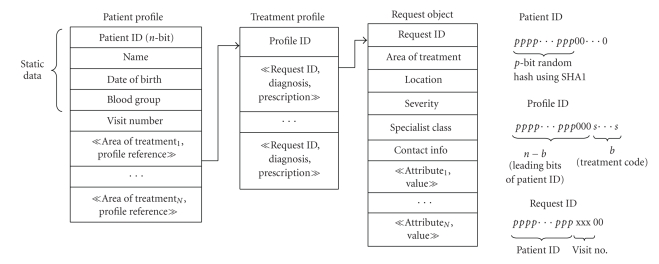
Structure of patient and treatment profiles, request object.

The telemedicine grid provides a persistent object-space abstraction, that is, the patient profile, treatment profile, patient request, and doctor profile, and so forth can be modeled as objects (inherited from a *Sharable* class). For instance, Figure 4 shows the structure of patient profile, treatment profile, and request object used in the telemedicine system (along with their id-generation mechanism for clustering). The patient profile stores general information about the patient and references to other treatment profiles (cardiology profile is one of such treatment profiles). Each treatment profile contains the treatment details for a particular area of treatment. For example, the cardiology profile will maintain references to patient request objects containing ECG along with the corresponding diagnosis and prescription. The patient request object is created each time the patient uses the telemedicine system. It captures the context information of the request and is used for scheduling. It also supports a generic (attribute name, value) field which holds the vital-parameter name and the corresponding vital-parameter measurement (or a pointer to it). The vital-parameter measurement could be a string or be stored in a file. For example, Blood Pressure value is a string, whereas ECG will be stored in a file.

#### 3.3.3. Clustering of Objects Per Patient

Clustering of objects per patient enables efficient retrieval of patient history. However, DHT-based storage mechanism does not support clustering (patient objects may be stored anywhere on the grid) because it uses consistent hashing techniques like SHA-1 for key generation. We modify the id-generation scheme in the telemedicine system as shown in Figure 4. This clusters the patient data and also ensures almost uniform distribution of objects over the object id space. Recall that prefix-based routing (Pastry) is used within the zone. In Pastry, two keys that differ only in the lower order bits (say *x*) will be stored within *x* hops of each other. In Figure 4, the patient profile object is assigned a random id (patient id) using SHA-1 (the last few bits are masked by zero). This ensures uniform distribution of patient profile objects on the DHT. The treatment profile ids are created by masking the last *b* bits of the patient id with the *treatment* code (every treatment area has a unique treatment code). This ensures that the patient profile objects and the treatment profiles are clustered. The patient request id is generated by inserting the visit number after the *p*-bit random patient id hash. This ensures that the request objects are spread out in comparison to the treatment profiles because their id prefix matches the patient id in fewer bits. This is the desired behavior since the doctor needs only aggregate history information available in the treatment profile while providing consultation. Thus queries for retrieving patient history can be answered efficiently with this clustering. Now, statistical variation in the node Ids and object Ids as well as variation in the object sizes (ex. high resolution images have greater size) can give rise to storage load imbalance. Hence, the telemedicine system incorporates replica diversion and file diversion schemes similar to that in PAST [24]. Details are given in [21].

### 3.4. Scheduling

#### 3.4.1. Objectives of Context-Aware Scheduling

Context is information that can be used to characterize the situation of the entity. Thus context-aware scheduling attempts to schedule the patient request to an appropriate doctor by considering context parameters like severity of ailment (normal/emergency), area of treatment, location of the patient, treatment history, language, and waiting time of the request. The scheduling mechanisms must handle normal as well as emergency situations in a timely manner by scheduling themto the nearest available doctor.The system must also improve the doctor's efficiency by reducing the system's response time. This calls for node capability-aware request placement, pre-fetching of patient history, client-side caching, and so forth are discussed in the following section.

#### 3.4.2. Operation of the Scheduler

The mobile doctors update their status (free/busy) through the mobile client application at the nearest hospital representative node. The hospital representative periodically publishes the doctors' availability information and a list of capable nodes at the zonal server. The size of the advertisement is less than 100 bytes and the number of hospitals per zone is of the order of 10^2^. Hence, the traffic overhead due to these advertisements is very less. The zonal server knows the location coordinates of every hospital within the zone. Hence, when a patient request arrives (containing the location coordinates, area of treatment) at the zonal server, it redirects the request to a capable node registered by a nearby hospital (having free request slots). Thereafter, the capable node accepts the patient request object and simultaneously fetches the patient history from the grid (DHT-lookup with the patient id). Next it composes the patient history and the request object into a single web page and locally hosts it. It also replicates the patient request object on the grid for fault-tolerance. At this point, it acknowledges the client application (patient side) with the patient request id, thereby completing the request upload process. Next, it provides the context parameters to the hospital representative which returns an appropriate doctor (doctor profile object is used for matching). It then notifies him with the object URL via email/SMS. Once the response is received, the request object is updated and the health worker is notified. Since all of the above are short-running tasks, it is reasonable to assume that the node remains capable and ensures best response time to the clients. We shall now look at the details of context-aware scheduling.

A patient request can be classified as emergency or normal, based on the value of the *“severity of ailment”* flag. The request must be immediately scheduled (preemptive way) if it is an emergency; or else some delay (from field study) is tolerable. Thus, based on the request type, the scheduler gets into an *emergency* mode or a *normal* mode. In the *emergency* mode, the request is immediately scheduled to more than one *ready specialist* (availability status = FREE). They could be idle or busy serving some request from the grid at that moment. The doctor-side application informs him of the emergency and allows him to preempt the current request and serve the emergency. If no doctor has downloaded the patient object within a threshold, then the request is rescheduled to double the number. 

**Figure 5:**
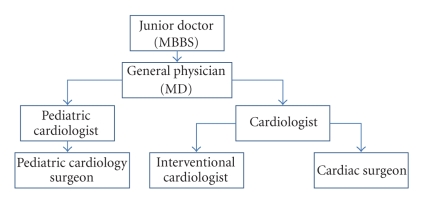
Hierarchy of specialists for heart ailment.

In the *normal* mode, scheduling mimics the day-to-day practice of hierarchical consultation. Figure 5 shows the hierarchy of specialists for heart ailment. This hierarchy is specified as an XML schema for every area of treatment. Thus the request is first scheduled to a nonspecialist. If he recommends further consultation, then the specialist class field of that request is set and the request is rescheduled. There can be two possible scenarios regarding the availability of doctors for scheduling.

1. Free doctors are available.
2. None of them are free.

Scenario 1 implies that the patient request can be immediately scheduled. In scenario 2, the request needs to be stored until a doctor becomes available. So the capable node inserts the request tuple (request id, age) into a location-wise tuple space. The *age* field indicates the request waiting time.

**(a) Realization of Location-Wise Tuple Space**

It is implemented on top of DHT (within the zone). The request tuple is inserted with a key equal to *hash* (location, area of treatment). The node to which the request tuple maps maintains a sorted list of tuples based on the *age *field. Now, every hospital polls the location-wise tuple spaces (of nearby locations) for any unfulfilled request with a frequency proportional to (number of requests ∗ average age of requests) that location. This ensures that too many requests do not get stacked at a particular location. Also it ensures that an aging request has a higher probability of getting fetched. If the request has aged beyond a threshold, then that request is made available globally (global tuple space). This also implies that a group of hospitals responsible for that location are busy. Thus, it acts as a hint to the zonal server to redirect requests from that location to some other hospital.

**(b) Realization of Global Tuple Space**

**Figure 6:**
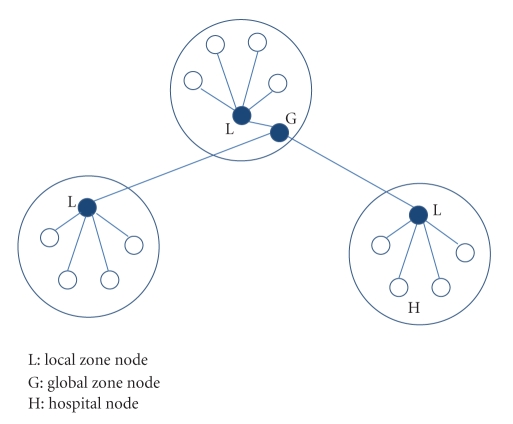
Realizing global space within and across zones.

Global tuple space is realized within and across zones for each area of treatment (e.g., Eye, heart, etc.). Each area of treatment is assigned a treatment code which is hashed using SHA-1 to produce a treatment hash. From every zone, the node whose id is the closest to the treatment hash holds all the aged request tuples for that area of treatment in that zone. We shall refer to it as local zone node id. Likewise, the zone whose id is the closest to the first *z* bits (*z* is the number of bits in the zone id) from the treatment hash is chosen as the global zone for that area of treatment. The node within that zone which is closest to the hash of the treatment hash (*rehashing ensures that the node chosen for global space within the zone and across zones is different*) stores the request load from all zones for that area of treatment. Henceforth, this node id will be referred as global zone node id. The local zone nodes advertise (zone id, request load) to the global zone node id if the number of pending requests is beyond a threshold. It is important to note that the request tuples are still retained within each zone and only the request load is stored at the global zone node id. Now, the zone having free request slots can query the global zone node id to find out the overloaded zones and serve their pending requests. But if every hospital node within a zone was to query the global zone node id, then it would become a hot spot. To handle this, the local zone node within each zone acts as a request collector. It accepts request subscriptions, that is, (hospital node IP, free request slots) from the hospital nodes. Periodically, it contacts the global zone node id to fetch the overloaded zone ids and then fetches the request ids from the corresponding local zone node id. Figure 6 shows the hierarchical realization of the global space.

**(c) Load Balancing of the Local Zone Node ID and Global Zone Node ID**

In the above scheme, the local zone node id and the global zone node id are potential bottlenecks. This problem can be alleviated by choosing multiple node ids distributed in the node id space as request collectors. We exploit the property that two keys (in Pastry) which differ in the higher order bits will be mapped to two different nodes that are wide apart in the node id space. We decide the number of leading bits (say *d*) based on the request load within the zone. Thus the request load is now shared by 2^*d*^ nodes within the zone. Similarly, when the request load goes down, a merge process is initiated and a new value of *d* is calculated. The information stored at the global zone node id viz. (zone id, d) does not change frequently and hence the global zone node replicates this information on its in-degree nodes. Thus queries from local zone nodes do not necessarily have to reach the global zone node and load balancing is achieved. Details of this scheme are available in [21].

### 3.5. Fault-Tolerance

Standby nodes (initially configured by administrator) are maintained to recover from zonal server failures while the grid node failures are handled as in Pastry and Chord DHTs. The state maintained by the hospital representative is replicated on the DHT. Details are available in [21].

### 3.6. Privacy, Confidentiality, and Security of Patient Data

The telemedicine report to Congress [25] defines Information Privacy as “the ability of an individual to control the use and dissemination of information that relates to himself or herself”. Privacy is ensured by maintaining the confidentiality of patient data, which means that specific controls are exercised on access and disclosure of patient data. *S*ecurity means protecting both the system and the information from unauthorized access (misuse and damage). In the context of Indian rural population (where 1 out of 6 dies in rural areas due to lack of health care facilities) saving lives is more important than the privacy of data. So, we provide very basic authentication schemes as part of the prototype. However, we outline one scheme which we would like to incorporate in the near future. At a higher level, the telemedicine grid preserves the anonymity of the patient as there is no direct interaction between the doctor and the patient. Instead, the interactions are patient-grid and grid-doctor (unless during emergencies). Besides that, the patient data visible to the doctor does not include patient identifiable information like his name, address, and so forth. It is only a 1024-bit identifier.

Minimally, the doctor, the health worker, and the patient are authenticated by the system before they can perform any operation. We now identify the vulnerabilities in the telemedicine grid system and outline a security scheme for maintaining the confidentiality of patient data. The patient data can be tampered/misused at the entry point (health worker), during transit, or by the doctor or within the grid system. The entry-point tampering is prevented by automating the entire process. Basically, the vital parameters captured by the electromedic devices are transferred via Bluetooth to the health worker PDA, thereby eliminating any manual entry. At this point the request data is encrypted using the patient's key (smart card). Next, the health worker's session key is used to calculate the request message hash and transfer it to the grid. This maintains the integrity of the patient request during transit (data transferred from the health worker device). Now we are trying to integrate proxy re-encryption schemes like in [26] into the system. In this scheme, the patient data in the system is securely locked with the patient's public key so that no intruder can access it. While access needs to be given to the selected doctor, the patient computes the *reencryption key* from his private key and the chosen doctor's public key. The grid uses this *reencryption key* to provide access to the doctor without compromising the patient's private key. There are many variants of the proxy reencryption schemes some of which can allow access to the doctor only for a certain period of time. Thus data privacy can be maintained in the telemedicine grid.

## 4. Application Scenario, Implementation, and Limitations

Our proposed telemedicine framework benefits both doctors and patients by improving their efficiency in terms of time, money and the medical consultation process. This nonvideo telemedicine framework works well for most cases (emergency/general consultations). We present a comparison of the existing medical system and the change brought about by introducing our model.

### 4.1. Existing Model

People in rural areas approach local doctors for diagnosis of an ailment. If local doctors (with limited knowledge) are not able to diagnose the problem, they direct the patient to visit a specialist in the city. This requires that the people from the rural area move to the city incurring huge cost and inconvenience. In most cases, the aliment would not be severe enough for an advanced medical procedure. As a result, the specialists' resources are underutilized while causing unnecessary costs for the patients. This inhibits the rural people from going to the city for further check-up and results in gross neglect of individual health. In case of emergency situations there is hardly any expertise available for rural people.

### 4.2. New Telemedicine Framework

A (mobile) health worker equipped with the basic medical kit is placed within the reach of rural masses. Now, the rural patient has the facility of remote consultation using our telemedicine grid where the details of the patient's records are made accessible for the volunteering doctors. Consultation charge can be obtained from the patient to be reimbursed between the doctor and other entities involved. But such an amount is considerably less than the costs incurred otherwise by the patient who visits a city doctor. On the other hand, forwarding the patient to the relevant doctor and following a hierarchy structure among the doctors result in optimal usage of their resources. In emergency cases the health worker contacts those doctors who are in emergency-response mode to take their advice in giving first-aid treatment to the patients. This is especially vital in case of heart attacks and accidents. The hospitals which contribute resources to the grid can reach patients who require high-end medical treatment through remote consultations, thus improving their capacity utilizations. Such a grid also provides the patient with a choice of services from multiple hospitals. The uniqueness of our system is in scalability, cost-effectiveness and reliability provided by using the grid. Grid technology improves the processes involved in providing telemedicine services while improving the scalability of the system and reducing the costs, which are the two critical factors in the success of a telemedicine system. 

Our plan is to introduce the telemedicine system in two phases. Phase I or pilot testing would mainly cater to rural areas, whereas Phase II would extend to urban areas and would even incorporate continuous monitoring of patient health using wearable devices. The scheduling approach proposed in this paper can facilitate context-aware discovery of doctors for each of the above cases. The end-to-end telemedicine system was demoed in CEBIT 2009 [27] (setup shown in Figure 1). The Arogyasree project is collaborative work with Professor Wilhelm Stork's team at the University of Karlsruhe, Germany. The team in Karlsruhe primarily works on the design of an ECG jacket. The jacket supports a Bluetooth interface that can transfer ECG data to the smart phone which can then upload it on the grid. This jacket can also be used for the continuous monitoring of a patient's ECG in a nonintrusive manner and without requiring hospitalization. Other vital parameter measuring devices like BP, and Blood Glucose are available as part of the Biocomfort kit [28]. Thus, ECG, BP, and Blood Glucose measurements are included in the current version of the telemedicine system.

The grid middleware is implemented in java and provides access to the patient records via web-based interface (Apache Web Server). The patient data is stored in the form of XML files that can be aligned as per the HL7 CDA architecture [29]. The system does not support real-time video conferencing and is based on a store and forward model. However, if the necessary infrastructure is in place at the rural centre, it may be supported externally to the system. The economy model needs to be worked out in detail. We are looking at microhealth insurance models on the health grid as one of the approaches. Integrating patient smart cards, easing the doctor registration, and so forth need to be addressed.

## 5. Performance Studies

**Figure 7:**
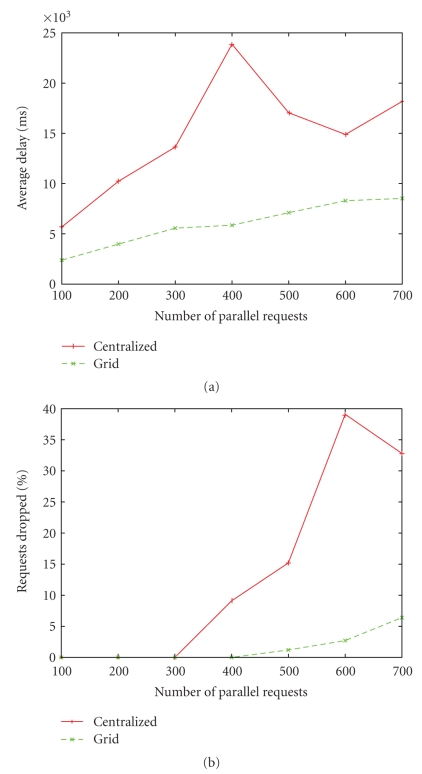
(a) Average delay (ms) versus number of parallel requests for centralized and grid scenarios, (b) requests drop (%) versus number of parallel requests for centralized and grid scenarios.

The data size of the objects for mobile telemedicine can at most be few MBs, because of constraints of bandwidth for the mobile device. In our case, the dual-channel ECGs captured for a period of 1 minute at 250 Hz frequency are approximately 64 KB size. We show a representative performance comparison by considering 750 KB sized objects. The experimental setup is as follows. The central server is a Quad Core with 4 GB RAM, while the grid comprises of 6 nodes that include P4s, Dual core machines with 1 GB RAM each. Parallel requests were made from 3 different machines. Figure 7(a) compares the average response time of the requests for the central server and the grid. The response time is much higher for the central server. Beyond 400 parallel requests, the response time slightly improves but the percentage of requests dropped increases as shown in Figure 7(b) (the connection time-out is set as 10 seconds and so the requests are dropped). Around 600 parallel requests, the percentage of dropped requests becomes more than 35% for the central server, whereas for the grid of 6 machines with 1 machine acting as the zonal server and redirecting the requests to the capable node, the failure rate is under 6 percent for even 700 requests. Clearly the grid solution of just 6 nodes performs better. 

**Figure 8:**
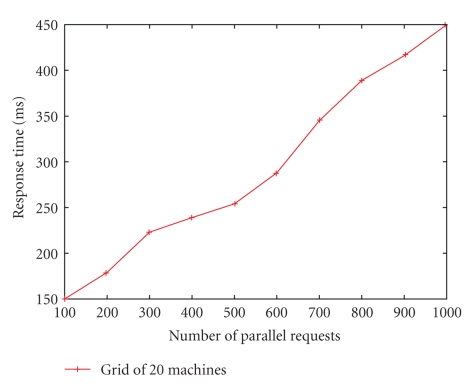
Response time (ms) for fetching patient history in a grid of size 20.

We deployed the telemedicine grid on 20 machines in our lab and measured the response time for fetching the patient history under different request loads (100–1000 parallel requests) with an average of 0.1 million objects per node (total = 2 million objects in the grid). The results are shown in Figure 8. The response time is less than 500 ms for 1000 parallel requests. It does not change significantly even if the average number of objects per node is increased to 0.5 million, since the grid is realized as a persistent object space and only the local disk access time increases with the number of objects stored. The DHT lookup time depends on the routing hops which is proportional to the log of the number of nodes in the overlay. For zone size of 1000, the average number of Pastry routing hops is 2.5 as shown in [23]. Since most of the requests are satisfied within the zone, the routing overhead is low. 

**Figure 9:**
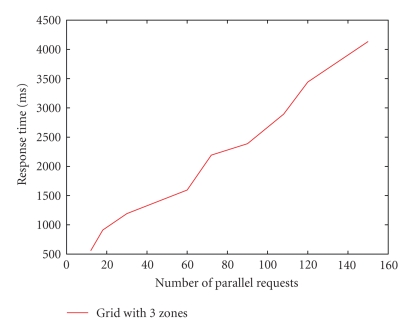
Response time (ms) for interzonal requests.

We have also measured the response time for the interzonal requests. For this experiment, we created 3 zones each having 6 nodes. Typically, a zone would span a city, and hence interzonal requests (across nearby cities) will incur an internet delay (typically ~50 ms). So we emulated the internet delay for interzonal request by setting it around 50 ms. Next, parallel requests are started from a zone for objects stored in other zone and the lookup times are measured. The average lookup time up to 150 parallel requests is shown in Figure 9. 

We observe that the average lookup time for interzonal requests is less than 5 seconds for up to 150 requests in parallel. Note that the interzonal routing occurs only when there are not enough resources in the zone. Such situations may arise during emergencies like epidemics when one zone gets affected.

## 6. Conclusion

In this paper, we proposed P2P architecture for mobile telemedicine. We showed that it is more scalable than a central server-based model. We also presented the design of a distributed context-aware scheduler (using tuple spaces) that considers parameters like proximity, patient history, severity of ailment, and so forth to locate appropriate doctors. The context-aware discovery of doctors can ensure timely fulfillment of requests and even reduce the cost. It becomes more important in the case of continuous monitoring when alerts need to be suitably addressed. Next we presented the zone-based overlay structure that ensures proximity in routing and storage. This zone-based architecture has been successfully used in our earlier work (Vishwa [9]) on P2P computational grids. Thus this architecture for a large-scale telemedicine grid will allow us to leverage the distributed resources for both data storage and computation, thereby aiding medical research communities. Our future work consists of conducting field trials, improving the user interfaces/security and integrating microhealth insurance models.
